# *In Vitro* Effects of Lead on Gene Expression in Neural Stem Cells and Associations between Up-regulated Genes and Cognitive Scores in Children

**DOI:** 10.1289/EHP265

**Published:** 2016-08-26

**Authors:** Peter J. Wagner, Hae-Ryung Park, Zhaoxi Wang, Rory Kirchner, Yongyue Wei, Li Su, Kirstie Stanfield, Tomas R. Guilarte, Robert O. Wright, David C. Christiani, Quan Lu

**Affiliations:** 1 Department of Environmental Health,; 2 Program in Molecular and Integrative Physiological Sciences, and; 3 Department of Biostatistics, Harvard T.H. Chan School of Public Health, Boston, Massachusetts, USA; 4 Department of Environmental Health Sciences, Columbia University Mailman School of Public Health, New York City, New York, USA; 5 Department of Preventative Medicine, Mount Sinai School of Medicine, New York City, New York, USA; 6 Department of Genetics and Complex Diseases, Harvard T.H. Chan School of Public Health, Boston, Massachusetts, USA

## Abstract

**Background:**

Lead (Pb) adversely affects neurodevelopment in children. Neural stem cells (NSCs) play an essential role in shaping the developing brain, yet little is known about how Pb perturbs NSC functions and whether such perturbation contributes to impaired neurodevelopment.

**Objectives:**

We aimed to identify Pb-induced transcriptomic changes in NSCs and to link these changes to neurodevelopmental outcomes in children who were exposed to Pb.

**Methods:**

We performed RNA-seq-based transcriptomic profiling in human NSCs treated with 1 μM Pb. We used qRT-PCR, Western blotting, ELISA, and ChIP (chromatin immunoprecipitation) to characterize Pb-induced gene up-regulation. Through interrogation of a genome-wide association study, we examined the association of gene variants with neurodevelopment outcomes in the ELEMENT birth cohort.

**Results:**

We identified 19 genes with significantly altered expression, including many known targets of NRF2—the master transcriptional factor for the oxidative stress response. Pb induced the expression of *SPP1* (secreted phosphoprotein 1), which has known neuroprotective effects. We demonstrated that *SPP1* is a novel direct NRF2 target gene. Single nucleotide polymorphisms (SNPs) (rs12641001) in the regulatory region of *SPP1* exhibited a statistically significant association (*p* = 0.005) with the Cognitive Development Index (CDI).

**Conclusion:**

Our findings revealed that Pb induces an NRF2-dependent transcriptional response in neural stem cells and identified SPP1 up-regulation as a potential novel mechanism linking Pb exposure with neural stem cell function and neurodevelopment in children.

**Citation:**

Wagner PJ, Park HR, Wang Z, Kirchner R, Wei Y, Su L, Stanfield K, Guilarte TR, Wright RO, Christiani DC, Lu Q. 2017. *In vitro* effects of lead on gene expression in neural stem cells and associations between up-regulated genes and cognitive scores in children. Environ Health Perspect 125:721–729; http://dx.doi.org/10.1289/EHP265

## Introduction

As a pervasive environmental toxicant, lead (Pb) particularly impairs the functions of the neural system (Bellinger 2013; Toscano and Guilarte 2005). While policy limiting the use of Pb has been successful in reducing blood Pb levels in U.S. children (Jones et al. 2009), Pb levels in the environment remain high in many countries where Pb has not—or has only recently—been phased out from gasoline, paint, and other applications. In the United States, more than half a million 1- to 5-year-old children still have blood Pb levels exceeding 10 μg/dL, twice the current threshold of concern defined by the Centers for Disease Control and Prevention (CDC) (Jones et al. 2009). Pb exposure in children has been consistently linked to impaired neurological development and cognitive dysfunction as well as persistent antisocial and delinquent behavior (Bellinger et al. 1987; Canfield et al. 2003; Needleman et al. 1996). Recent incidences of Pb contamination in drinking water in several U.S. cities highlight the continued threat of Pb to public health, especially to children’s health (Bellinger 2016; Levin 2016).

Pb neurotoxicity is determined by intricate interplays between the metal and target neural cells, and there is overwhelming evidence documenting the detrimental effects of Pb in neurons. Seminal studies by Alkondon et al. (1990) and Guilarte and Miceli (1992) showed that Pb potently inhibits the NMDA receptor, which plays an essential role in brain development, synaptic plasticity, and learning and memory. Pb also inhibits the vesicular release of BDNF (brain-derived neurotrophic factor) and subsequent TrkB (tropomyosin-related kinase B) activation in the presynaptic neuron (Neal et al. 2010, 2011; Stansfield et al. 2012).

Pb exposure at the early stages of brain development has long-lasting effects on neurocognitive function. Prenatal Pb exposure has been associated with lower mental development index scores (Bellinger et al. 1987; Hu et al. 2006), and increased risk of schizophrenia later in life (Opler et al. 2004, 2008). In predictive models of mental developmental index (MDI), first trimester Pb exposure assessed in maternal blood was the most pronounced and statistically significant predictor when compared to exposure at any later stages (Hu et al. 2006). The particular susceptibility of early brain development to Pb exposure may be explained, in part, by the metal’s effects on neural stem cells (NSCs). As the progenitor cells of all cell types of the central nervous system, NSCs play an essential role in shaping the developing brain and could very well be affected by Pb exposure. Indeed, several studies have shown that Pb slows proliferation of NSCs both *in vitro* (Breier et al. 2008; Huang and Schneider 2004) and *in vivo* (Breier et al. 2008; Gilbert et al. 2005; Schneider et al. 2005; Verina et al. 2007), and alters gene expression to affect neuronal differentiation of mouse (Sánchez-Martín et al. 2013) and human stem cells (Senut et al. 2014).

Despite the known detrimental effects of Pb in NSCs, the underlying molecular mechanisms remain poorly understood, and moreover, whether such effects contribute to the impaired neurodevelopment in children is not known. In this study, we performed global transcriptional profiling to assess the impact of Pb exposure on NSCs. We characterized one of the gene hits, *SPP1* (also known as osteopontin), as a novel NRF2 target and determined whether genetic polymorphisms within the gene are associated with neurological outcomes in children in an epidemiological cohort. We integrated global gene expression profiling with genetic epidemiology and identified a potential mechanistic link between Pb-induced gene expression in NSCs and neurodevelopment in children.

## Materials and Methods

### NSC Culturing, Pb Treatment, and siRNA Transfection

NSCs derived from NIH-approved H9 (WA09) human embryonic stem cells were purchased from Life Technologies and cultured according to the supplier’s protocol. An aqueous solution of 1 mM Pb acetate trihydrate (cat. no. 316512; Sigma Aldrich) stock was used in all experiments. Transfection of siRNAs was performed with DharmaFECT 1 (ThermoFisher) following the manufacturer’s protocol. All siRNAs were obtained from Sigma—nontargeting control (SIC001), si-NRF2-1 (SASI_Hs01_00182393), si-NRF2-2 (SASI_Hs02_00341015), and si-KEAP1 (SASI_Hs01_00080908). All experiments were performed in passage 3 cells.

### Cell Viability and Growth Assays

For the MTT [3-(4,5-dimethylthiazol-2-yl)-2,5-diphenyl tetrazolium bromide] assay, cells were seeded 24 hr prior to exposure at 1 × 10^4^ per well of a 96 well plate. Exposure to 0, 0.5, 1, 2, 5, and 10 μM Pb was performed in 8 replicate wells. The assay was performed according to the MTT manufacturer’s protocol (Sigma Aldrich). Briefly, 0.05 mg of MTT was added to each well for 3 hr. Formazan crystals were solubilized in 10% Triton™ X-100 plus and 0.1 N HCl in anhydrous isopropanol after repeated pipetting. Absorbance was read at 570 nm, and background at 690 nm removed. Mean absorbance, which is correlated with cell number, is reported along with the standard error of the mean of eight replicate samples. For growth assay, human neural stem cells (hNSCs) were seeded in 24 well plates at 5 × 10^4^ per well and treated with the control vehicle phosphate-buffered saline (PBS) or human recombinant SPP1 protein (Eton Bioscience) at 50 or 250 ng/mL. The next day, hNSCs were exposed to 2 μM Pb for 3 days. Cell counting was done by hemocytometer with Trypan blue staining to exclude dead cells. Six replicates were done for each condition.

### RNA-seq Library Preparation and Sequencing

Poly-adenylated RNA species were isolated from 1 μg of total RNA and converted to a cDNA library for RNA sequencing using the TruSeq® RNA v2 kit (Illumina). Sample preparation involves isolating poly-adenylated RNA, RNA fragmentation, cDNA synthesis, ligation of adapters, PCR amplification using DNA barcodes, and library validation and quantification. Four samples were multiplexed into a single lane of the Illumina HiSeq 2000 for paired-end reads of 100 bp. Sequencing was performed at the Bauer Core Illumina Sequencing Facility (FAS Center for Systems Biology, Cambridge, MA).

### Processing and Analysis of RNA-Seq Data

Low-quality reads (< 25 phred), adaptors, and poly-A tails were trimmed with cutadapt (Martin 2011). Read pairs with one or more reads shorter than 20 bp were removed. Quality of reads was assessed using FASTQC (Babraham Bioinformatics). Reads were aligned to human genome build 19 using Tophat2 (Trapnell et al. 2009) and compiled into count tables using HTseq-count (International HapMap Consortium 2003). Counts were normalized in edgeR (Robinson and Oshlack 2010). Differential expression was determined by a generalized linear model. Differentially regulated transcripts were identified following a Benjamini–Hochberg multiple testing correction (*q* < 0.05) that had a greater than ± 0.2 fold change and a minimum count per million mapped of one.

### qRT-PCR

RNA was reverse transcribed using SuperScript™ III reverse transcriptase and oligo-dT (Life Technologies). The resulting cDNA was amplified using 2× SYBR® mix (Qiagen) and 3 mM of each primer in a StepOne Plus Thermocycler (Applied Biosystems) in quantitative reverse transcriptase polymerase chain reaction (qRT-PCR). Melt curves were checked for single-length amplification products. Fold changes were calculated using the 2-ΔΔCt method. GAPDH is the housekeeping gene used for normalization in all qPCR assays. All primers used in this study and their respective sources or design are listed in Table S1.

### SPP1 Western Blotting and ELISA

SPP1 levels were assessed in whole cell extract using standard Western blotting procedures with 1:1,000 Anti-Osteopontin antibody (EPR3688; abcam). Relative protein concentrations were quantified in Image-J (NIH; see https://imagej.nih.gov/ij/). SPP1 levels in cell culture media was assessed using the human osteopontin (OPN) Quantikine® ELISA Kit (DOST00; R&D Systems). Media was sampled 60 hr post-transfection, after 48 hr of contact with the cells.

### Chromatin Immunoprecipitation (ChIP) Assay

NSCs were expanded to approximately 8 million cells, of which half were enriched for NRF2 using siRNA knockdown of NRF2’s negative regulator KEAP1 and the other half were transfected with a nontargeting siRNA control. Samples were prepared following the SimpleChIP® Plus Enzymatic Chromatin IP Kit protocol supplied by the manufacturer (Cell Signaling Technology). Briefly, 48 hr post-transfection, NRF2 was cross-linked to DNA using 1.5% formaldehyde. Nuclei were collected and lysed by sonication. Chromatin DNA was digested with micrococcal nuclease for 18 min into small fragments (150–900 bp). Nuclear extracts were incubated overnight with NRF2 antibody (cat. no. 12721; Cell Signaling Technology) and antibody-bound complexes were captured by SureBeads™ protein G magnetic beads (Bio-Rad). Bound DNA was purified and underwent quantitation by PCR using primers for putative SPP1 ARE, NQO1 ARE (Chorley et al. 2012) and RPL30-exon 3 (Cell Signaling Technology).

### The ELEMENT Cohort, Pb Exposure, and Neurodevelopmental Indexes

The cohort of infants analyzed in this study is a subset of the Early Life Exposure in Mexico to ENvironmental Toxicants (ELEMENT) prospective birth cohort, which was designed to assess the roles of environmental and social stressors in birth outcomes as well as infant and child development. The characteristics of the cohort are provided in Table S2. Between 2007 and 2011, mothers were recruited during pregnancy and only one child for each mother was included in the study. Relevant to this study, prenatal Pb exposure was assessed from maternal blood during the second trimester of pregnancy. Infant neurodevelopment was assessed at 24 months of age using a Spanish version of the Bayley Scales of Infant and Toddler Development, Third Edition (Bayley 2005). Three primary outcome indices are derived from the assessment: the Cognitive Development Index (CDI), the Language Development Index (LDI) and the Psychomotor Development Index (PDI). Detailed information on the study design and data collection procedures have been published previously (Ettinger et al. 2009; González-Cossío et al. 1997; Hernandez-Avila et al. 2002). The human subjects committees of the National Institutes of Public Health in Mexico, Harvard T.H. Chan School of Public Health, Icahn School of Medicine at Mt. Sinai, and participating hospitals approved all study materials and procedures. Women and children who were old enough signed informed consent letters before enrollment. Consent obtained at enrollment applies to the research described in this study.

### Prenatal Pb Exposure Assessed in Second Trimester Maternal Blood

In the second trimester of each expectant mother, maternal venous blood was collected in trace element-free tubes and frozen. Samples were shipped at 4°C to the Trace Metals Laboratory at the Harvard T.H. Chan School of Public Health, Boston, MA. Samples were processed in a dedicated trace metal clean room outfitted with a Class 100 clean hood using glassware cleaned for 24 hr in 10% HNO3 and rinsed several times with 18Ω Milli-Q water. Approximately 1 g of blood from each mother was digested in 2 mL concentrated nitric acid for 24 hr, and subsequently overnight in 30% hydrogen peroxide (1 mL per 1 g of blood). Samples were diluted to 10 mL with deionized water. Acid-digested samples were analyzed for total Pb using dynamic reaction cell inductively coupled plasma mass spectrometry (DRC-ICP-MS, Perkin Elmer). Final values are the average of five replicate measurements for each individual sample.

### Assessment Using the Bayley Scales of Infant and Toddler Development

Infant neurodevelopment was assessed at 24 months of age using a Spanish version of the Bayley Scales of Infant and Toddler Development (Bayley 2005). Three primary outcome indices were derived from the assessment: the CDI, a composite variable of test scores pertaining to cognition; the LDI, a composite variable of test scores pertaining to expressive and receptive language; and the PDI score, a composite variable of test scores pertaining to fine and gross motor skills.

### Genome-wide Genotyping Using an Illumina SNP Chip

DNA was extracted from umbilical cord blood samples in the ELEMENT cohort using the Puregene DNA isolation kit (Gentra Systems) and stored at –20°C. Samples were genotyped using the high density Illumina 1 Million Duo chip at the Center for Applied Genomics of the Children’s Hospital of Philadelphia.

### SNP Analyses for Main Effect and Pb Interaction

Following the quality control assessment, genotypes of 16 SNPs within the *SPP1* transcribed locus or within the 10 kb flanking regions were available for 462 infants in the ELEMENT cohort. Linear regression analyses for both main effect and interaction were performed using PLINK (version 1.07) (Purcell et al. 2007). For main effect, linear regression analyses of minor allele copy number on the three outcomes (CDI, LDI, and PDI) were adjusted for sex, gestational age, maternal age, marital status, presence of siblings, maternal education (high school vs. no high school), age at Bayley Scale assessment, and genome-wide principal components 1 and 2. For interaction analyses, natural log transformation of second trimester maternal blood Pb and a multiplicative interaction term of minor allele copy number and natural log transformation of second trimester maternal blood Pb were included in regression analyses. The *p*-value cut off for statistical significance was determined using the method proposed by Li and Ji (2005), which takes into account that each SNP test is not an independent test given the linkage disequilibrium (LD) among neighboring SNPs. An LD map and haplotypes were generated using the genotyped data using LD-Plus (Vanderbilt University).

## Results

### RNA-seq Identified Gene Expression Changes in Pb-treated NSCs

**Figure 1:**
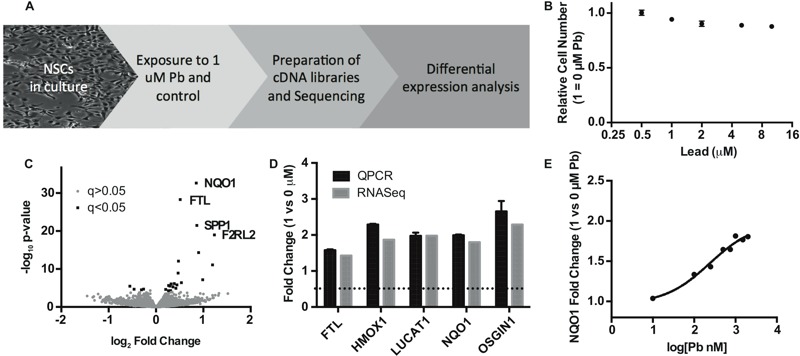
Identification of differential gene expression in human NSCs exposed to Pb by RNA-seq. (*A*) Schematic workflow of the study. (*B*) MTT assay showing relative numbers of NSCs after 24 hr treatment of Pb at different concentrations. Error bars represent standard error of the mean of eight replicates. (*C*) Volcano plot of RNA-seq results with top four genes annotated. Black squares represent differentially expressed genes defined by > 0.2 log_2_-fold change and FDR-adjusted *q*-value < 0.05%; gray circles represent genes that do not meet the significance threshold. (*D*) qPCR validation of known NRF2 targets identified by RNA-seq. Results were obtained from three biologic replicates. (*E*) Induction of *NQO1* expression in response to 24-hr Pb treatment (at different concentrations) in NSCs.

To better understand the effects of Pb on NSCs, we performed global transcriptional profiling in human NSCs exposed to Pb. We chose to use RNA sequencing (RNA-seq) as we reasoned that the sensitivity of the method might allow for the identification of subtle yet significant changes in gene expression. Because we were particularly interested in the effect of Pb on prenatal neurodevelopment, we used human NSCs that were initially generated from an embryonic stem cell line (H09 line). We exposed cultured human NSCs to Pb or vehicle control (Figure 1A). The Pb concentration used in this study (1 μM, or 20.7 μg/dL) is about four times the current CDC level of concern for blood Pb and is within the range in exposed human populations (Pirkle et al. 1994; Zheng et al. 2008). Exposure of NSCs cells to 1 μM Pb for 24 hr resulted in a slight decrease (5%) in cell number compared to that in the control vehicle-treated cells (Figure 1B). This is consistent with previous studies showing the inhibitory effect of Pb on NSC proliferation (Breier et al. 2008; Huang and Schneider 2004).

Using total RNAs from control and Pb-treated NSCs, we constructed RNA-seq libraries, each with a unique barcode that allows multiplexing. To minimize the variation of sequencing runs, we pooled barcoded RNA-seq libraries for next generation deep sequencing. We obtained an average of ~ 38 million reads per sample and tested for differential expression of GRCh37 Ensembl-annotated genes. Following a stringent Benjamini–Hochberg multiple testing correction (α < 0.05), we identified a total of 19 differentially expressed genes (3 down-regulated and 16 up-regulated) in Pb-treated NSCs, as shown in Table 1 and in the volcano plot in Figure 1C. Full results from the differential expression analysis are included in Excel File Table S1.

**Table 1:** Differential expression of human NSCs exposed to 1 μM Pb by RNA-seq.

HGNC name	Gene description	Fold change	*p*-Value	FDR *q*-value	NRF2 target
*F2RL2*	Coagulation factor II (thrombin) receptor-like 2	2.35	1.05 × 10^–19^	4.62 × 10^–16^
*OSGIN1*	Oxidative stress induced growth inhibitor 1	2.29	8.21 × 10^–12^	2.06 × 10^–8^	a
*LUCAT1*	Lung cancer associated transcript 1 (nonprotein coding)	1.98	6.60 × 10^–8^	1.16 × 10^–4^	g
*HMOX1*	Heme oxygenase (decycling) 1	1.87	4.63 × 10^–15^	1.63 × 10^–11^	a,b,d,e
*SPP1*	Secreted phosphoprotein 1	1.82	3.27 × 10^–22^	1.92 × 10^–18^
*NQO1*	NAD(P)H dehydrogenase, quinone 1	1.8	2.24 × 10^–33^	3.93 × 10^–29^	a,b,c,f
*EGF*	Epidermal growth factor	1.45	4.07 × 10^–7^	6.50 × 10^–4^
*FTL*	Ferritin, light polypeptide	1.43	5.00 × 10^–29^	4.39 × 10^–25^	a,b
*VGF*	VGF nerve growth factor inducible	1.39	8.65 × 10^–13^	2.53 × 10^–9^	b
*TXNRD1*	Thioredoxin reductase 1	1.38	1.22 × 10^–9^	2.68 × 10^–6^	a,b,f
*SERPINE1*	Serpin peptidase inhibitor, clade E (nexin, plasminogen activator inhibitor type 1), member 1	1.36	1.53 × 10^–6^	1.92 × 10^–3^	b,c,f
*SLC7A11*	Solute carrier family 7 (anionic amino acid transporter light chain, xc- system), member 11	1.34	1.20 × 10^–5^	1.11 × 10^–2^	a
*SLC7A8*	Solute carrier family 7 (amino acid transporter light chain, L system), member 8	1.31	6.88 × 10^–7^	1.01 × 10^–3^
*GREM1*	Gremlin 1, DAN family BMP antagonist	1.28	5.19 × 10^–6^	5.07 × 10^–3^
*PIR*	Pirin (iron-binding nuclear protein)	1.25	8.54 × 10^–7^	1.15 × 10^–3^	a
*F13A1*	Coagulation factor XIII, A1 polypeptide	1.24	5.05 × 10^–5^	3.55 × 10^–2^
*B3GALT2*	UDP-Gal:betaGlcNAc beta 1,3-galactosyltransferase, polypeptide 2	0.81	3.00 × 10^–5^	2.29 × 10^–2^
*MIR503HG*	MIR503 host gene (non-protein coding)	0.73	2.14 × 10^–5^	1.79 × 10^–2^
*DIO3OS*	DIO3 opposite strand/antisense RNA	0.68	3.35 × 10^–6^	3.47 × 10^–3^
Differential expression of triplicate pairs was performed in edgeR, and statistically significant differentially expressed transcripts were defined by > ± 0.2 log_2_-fold change and FDR-adjusted *q*-value < 0.05%. Annotation shows known NRF2 target genes according to a) Chorley et al. (2012), b) Wang et al. (2007), c) Cho et al. (2005), d) Lee et al. (2003), e) Li et al. (2002), f) Malhotra et al. (2010), and g) Thai et al. (2013).					

### Effects of Pb on NRF2 Target Gene Expression

Among the most statistically significant up-regulated genes in Pb-treated NSCs are *NQO1* and *HMOX1*, which are well known targets involved in the cellular response to oxidative stress. The cellular oxidative stress response is mediated by the master transcriptional factor NRF2 (Kensler et al. 2007). NRF2 works to activate transcription by binding to the antioxidant responsive elements (AREs) in target genes. Many genes in addition to *NQO1* and *HMOX1* also contain ARE elements and are targets of NRF2. We thus examined the rest of the Pb-up-regulated genes and found that at least 10 out of 16 genes had been previously identified as direct targets of NRF2 (Table 1). The expression changes of many of these genes induced by Pb were confirmed by qPCR. As shown in Figure 1D, there is a remarkable consistency between the levels of gene expression measured by RNA-seq and qPCR. The induction of *NQO1*, *HMOX1* and the other known NRF2 target genes strongly suggests that Pb elicits oxidative stress and activates NRF2 in NSCs. Using *NQO1* expression as a surrogate marker for NRF2 activation, we determined the dose response of NSCs to Pb. As shown in Figure 1E, Pb as low as 0.1 μM induced a significant increase (33%) in *NQO1* expression, indicating that NRF2 activation may be particularly sensitive to Pb exposure in NSCs.

### Effect of Pb on SPP1 Expression

**Figure 2:**
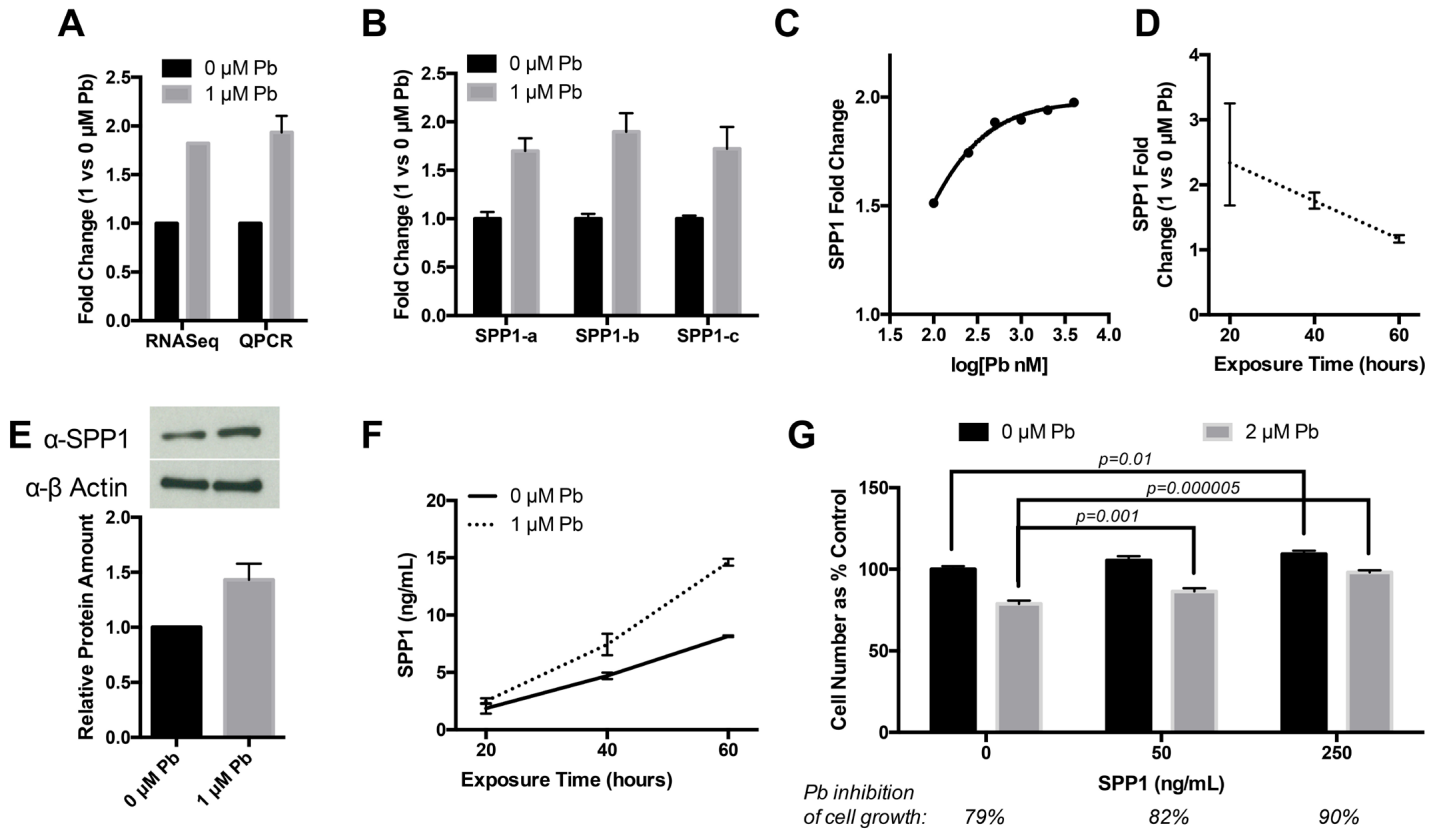
Effects of Pb treatment on SPP1 expression in NSCs. (*A*) Comparison of *SPP1* induction-fold changes measured by qPCR and RNA-seq. (*B*) qPCR of three major *SPP1* splice variants after 24-hr Pb exposure. Splice-variant specific primers were used. (*C*) Dose–response curve of SPP1 exposed to a range of Pb concentrations for 24 hr. (*D*) Time course of SPP1 exposed to 1 μM Pb. (*E*) Upper panel: SPP1 Western Blotting of whole cell extracts from control or 24-hr Pb-treated NSCs. Lower panel: relative SPP1 protein amount normalized against β-actin. (*F*) Cell culture media concentration of SPP1 determined by ELISA in control or Pb-treated NSCs (for 20, 40 and 60 hr). All error bars represent the standard error of the mean of three biologic replicates. (*G*) hNSCs seeded in 24 well plates at 5 × 10^4^ per well were treated with control vehicle PBS or human recombinant SPP1 protein (Eton Bioscience) at 50 or 250 ng/mL. The next day, hNSCs were exposed to 2 μM Pb for 3 days. Cell counting was done by hemocytometer with Trypan blue staining to exclude dead cells. Six replicates were done for each condition.

Four genes (*SPP1*, *F2RL2*, *EGF*, and *SLC7A8*) that were not previously known as NRF2 targets are up-regulated by Pb in NSCs (Figure 1C and Table 1). SPP1, also known as OPN, is an extracellular matrix protein that has been shown to be up-regulated in neuro-injury and is implicated as a potent neuroprotectant (Meller et al. 2005; Topkoru et al. 2013). Because of its potential connection to neural function, SPP1 was chosen for the follow-up characterization. The up-regulation of SPP1 by Pb in NSC cells was confirmed by qRT-PCR (Figure 2A). The *SPP1* gene has three splice variants, all of which were up-regulated upon Pb exposure (Figure 2B), indicating that the mechanism involved in *SPP1* up-regulation by Pb is not splicing variant specific. Comparison of Ct values indicates *SPP1-A* is the dominant form in NSCs, with mRNA levels ~ 10-fold higher than *SPP1-B* and ~ 20-fold higher than *SPP1-C* (data not shown). Dose response showed that Pb induced SPP1 mRNA expression at 0.1 μM and that the effect maximized at around 2 μM (Figure 2C). The extent of *SPP1* induction by Pb was higher at 20 hr than at later time points (Figure 2D), suggesting a potential negative feedback regulation of *SPP1* mRNA expression. Consistent with mRNA up-regulation, Western blotting showed that total SPP1 protein level is increased in Pb-treated NSCs (Figure 2E). Since SPP1 is a secreted protein, we measured the amount of SPP1 protein in the culture medium of Pb-exposed and unexposed NSCs. As shown in Figure 2F, there was more SPP1 protein in the media of NSCs cell culture of Pb-exposed NSCs than in the control cells. After 60 hr of Pb exposure, the level of SPP1 in culture media of Pb-exposed cells was 1.8-fold higher than in unexposed cells.

Studies have shown that SPP1 is pro-proliferative (Kalluri and Dempsey 2012) and mediates the survival and proliferation of neural stem cells (Rabenstein et al. 2015). SPP1 up-regulation by Pb thus may constitute a mechanism to protect NSCs from Pb toxicity. We have shown that Pb inhibits hNSC proliferation (Figure 1B). We thus determined whether SPP1 attenuates the inhibitory effect of Pb on hNSCs proliferation. As shown in Figure 2G, addition of recombinant human SPP1 protein at 250 ng/mL in the culturing medium significantly increased the growth of hNSCs in the presence of Pb (90% vs. 79%). Even at lower concentration (50 ng/mL), SPP1 still increased hNSC cell growth in Pb-treated hNSCs, though to a lesser extent. These data support a neuroprotective role of SPP1 in reducing Pb toxicity in hNSCs.

### The Role of NRF2 in Pb-induced *SPP1* Up-regulation

**Figure 3:**
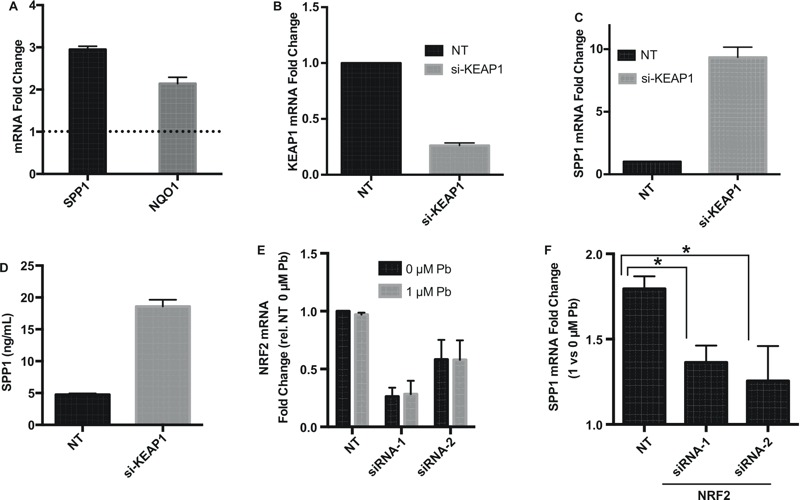
NRF2-dependent up-regulation of SPP1 expression. (*A*) *SPP1* and *NQO1* expression-fold changes (measure by qPCR) in NSCs exposed to canonical NRF2 inducer DL-Sulforaphane (DLS, 1 μM) for 24 hr. (*B*) Efficiency of siRNA knockdown of KEAP1 48 hr post transfection as assessed by qPCR. NT: nontargeting control siRNAs. (*C*) *SPP1* expression in control or KEAP1 knockdown NSCs. qPCR was done 48 hr post KEAP1 siRNA transfection. (*D*) Amount of secreted SPP1 protein in cultured media of NSCs at 60 hr post KEAP1 siRNA transfection. (*E*) Efficiency of siRNA knockdown of NRF2 using two siRNAs 48 hr post transfection. Pb and vehicle control were added to cells 24 hr post transfection for a 24-hr exposure. (*F*) SPP1 expression after 24 hr of Pb exposure in NRF2-knockdown cells compared to that in NT-siRNA transfected cells. All figure error bars represent standard error of the mean of three biologic replicates.

We next determined whether *SPP1* up-regulation by Pb in NSCs is part of the NRF2-mediated oxidative stress response. We exposed NSCs to the canonical NRF2 activator DL-Sulforaphane. As shown in Figure 3A, 1 μM DL-Sulforaphane significantly induced the expression of *NQO1* and *SPP1* expression. NRF2 is normally sequestered and degraded in the cytoplasm by its negative regulator KEAP1 (Kensler et al. 2007). Upon oxidative stress, NRF2 dissociates from KEAP1, accumulates and then translocates to the nucleus. Thus, NRF2 can be activated by inactivation of KEAP1. A pooled siRNA-mediated knocked down of KEAP1 by > 70% (Figure 3B) led to an increase in both *SPP1* mRNA (9.6-fold 48 hr post siRNA transfection) and in the secreted SPP1 protein (3.9-fold, 60 hr post siRNA transfection) (Figure 3C,D). Together, these results indicate that NRF2 activation leads to increased *SPP1* expression.

To test whether NRF2 is required for *SPP1* up-regulation, we knocked down NRF2 in NSCs and subsequently subjected the NSCs to Pb treatment. As shown in Figure 3E, two siRNAs both efficiently knocked down *NRF2*. Pb exposure itself did not affect either the baseline NRF2 expression or the knockdown efficiency (Figure 3E). However, knockdown of NRF2 significantly attenuates Pb-induced *SPP1* up-regulation (Figure 3F). The effect is likely specific as two different NRF2 siRNAs produced similar attenuation on *SPP1* up-regulation. These data indicate that up-regulation of *SPP1* by Pb exposure in NSCs is mediated by NRF2.

### Direct Transcriptional Regulation of *SPP1* by NRF2

**Figure 4:**
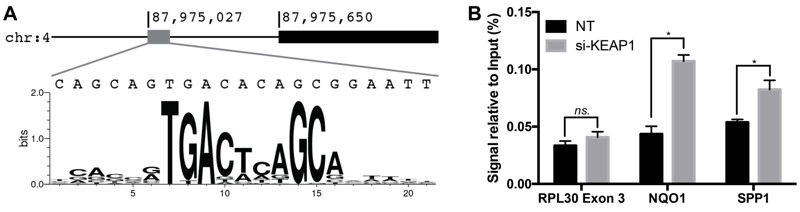
NRF2 interaction with an ARE in the SPP1 promoter. (*A*) Presence of a putative ARE ~ 600 bp upstream of SPP1 transcription start site. (*B*) NRF2 ChIP followed by PCR amplification of the putative SPP1 ARE. NRF2 was activated by KEAP1 knockdown and NT-siRNA was transfected into control cells. Following NRF2 ChIP, qPCR was done to measure the presence of SPP1 ARE, NQO1 ARE (positive control), and RPL30 Exon 3 (negative control). *p < 0.05 by two-sided *t*-test.

NRF2 controls target gene expression by binding to specific DNA sequences known as AREs within the promoters of target genes. Analyses of AREs in the promoters of canonical NRF2 target genes have identified a consensus sequence motif (RTKAYnnnGCR) that is required for NRF2 binding (Erickson et al. 2002). Using an ARE position weight matrix (Wang et al. 2007), we examined the promoter of *SPP1* and identified a putative ARE sequence ~ 600 bp up-stream of the transcription start site (Figure 4A). To test whether NRF2 directly interacts with the putative *SPP1* ARE, we performed a ChIP assay. We used KEAP1 knockdown to increase the NRF2 signal in NSCs. The NRF2 protein was immunoprecipitated and bound DNA fragments were examined to detect the presence of the putative *SPP1* ARE sequence. We compared the signal level in control and KEAP1-knockdown cells. As shown in Figure 4B, the qPCR signal specific to the *SPP1* ARE in KEAP1-knockdown NSCs was significantly higher than that in control NSCs. A similar increase was also observed for a canonical ARE located up-stream of *NQO1*, whereas no significant increase was observed for a non-NRF2 target sequence, *RPL30-exon 3*. In addition, control ChIP using rabbit IgG showed very little pull down of SPP1 ARE and there was no difference between KEAP1-knockdown and control cells (see Figure S1). Together these data indicate that the *SPP1* gene contains a functional ARE and is a direct target of NRF2.

### Association of SPP1 Genetic Polymorphism with Cognitive Development

**Figure 5:**
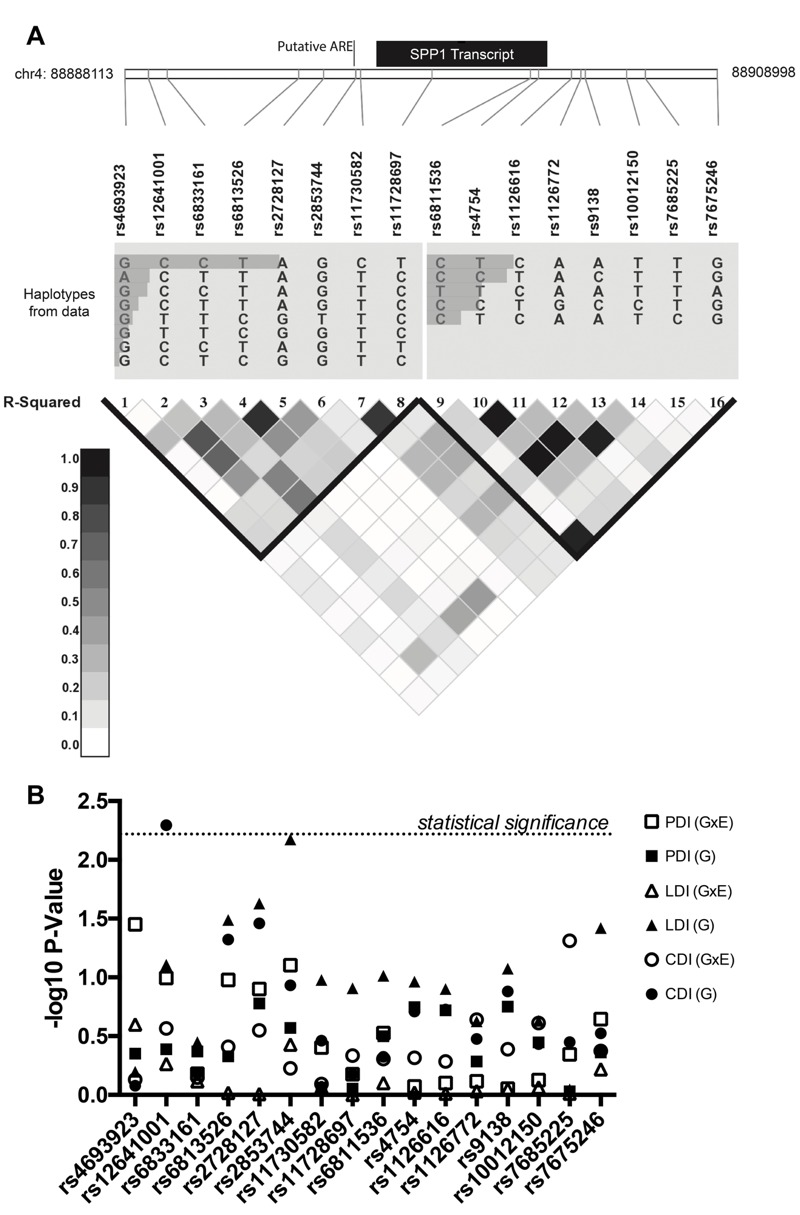
Association analysis of SPP1 SNPs with neurodevelopment phenotypes. (*A*) Schema of SNPs in relation to the* SPP1* transcribed locus and ARE. Linkage disequilibrium (LD) patterns around *SPP1* in the study population are shown. The relative abundances of haplotypes are shown above the LD plot. LD plot reflects pairwise *R* ^2^ among SNPs. (*B*) Negative log_10_ *p*-values for SNP associations with the Cognitive Development Index (CDI), Language Development Index (LDI) and Psychomotor Development Index (PDI) as a main effect (*G*) and in interaction with maternal second trimester lead (G × E).

Because NSCs play an important role in early brain development, we directly examined the association of SPP1 genetic variants with neurodevelopmental outcomes affected by Pb exposure in children. We took advantage of existing genotyping data from genome-wide association studies (GWAS) in the ELEMENT cohort (Z.W., unpublished data, 2016), which was designed to assess the roles of environmental and social stressors in birth outcomes. A total of 16 common *SPP1* SNPs (minor allele frequency > 5%) were genotyped in the ELEMENT cohort (*n* = 462). The relative genomic location and LD of the SNPs are shown in Figure 5A. We performed regression analyses to examine the main effect of the SNPs on the three cognitive outcomes: CDI, LDI, and Psychomotor Development Index (PDI), as well as the SNP interaction with Pb exposure (Table 2). From these analyses, we identified the SNP rs12641001 with a statistically significant main effect association with CDI (*p* = 0.005) (Figure 5B). Rs12641001 does not show a statistically significant interaction with second trimester Pb exposure. According to the model each copy of the minor allele, T, increases CDI by 2.6 points. Upon examining the LD map (Figure 5A), we found the T-allele of rs12641001 tags two haplotypes that spans the SPP1 promoter region and the first part of the transcript. One of the tagging SNPs (rs2728127) is suggestively associated with CDI with a raw *p*-value of 0.03471. Rs12641001 is suggestively associated with LDI (*p* = 0.078) but has no evident association with PDI (*p* = 0.4089). Therefore, rs12641001 has the largest and most significant association with CDI in children (Figure 5B).

**Table 2:** Summary of SPP1 SNP association effect sizes and *p*-values in determining CDI, LDI and PDI.

rs id	chr4 bp	Alleles	MAF	Cognitive development index (CDI)						Language development index (LDI)						Psychomotor development index (PDI)					
				Main effect		Interaction				Main effect		Interaction				Main effect		Interaction			
				β	*p*-Val	SNP		SNP*logPb		β	*p*-Val	SNP		SNP*logPb		β	*p*-Val	SNP		SNP*logPb	
						β	*p*-Val	β	*p*-Val			β	*p*-Val	β	*p*-Val			β	*p*-Val	β	*p*-Val
rs4693923	88888113	A/G	0.11	–0.18	0.8338	–0.65	0.6881	0.12	0.7425	0.44	0.6344	–1.16	0.5047	0.45	0.2516	–0.77	0.4461	–3.96	0.03735	0.91	0.03547
rs12641001	88888940	T/C	0.10	2.56	**0.00508**	3.97	0.01465	–0.37	0.2723	1.72	0.078	2.44	0.1589	–0.22	0.5449	0.88	0.4089	3.82	0.04506	–0.65	0.1012
rs6833161	88889605	T/C	0.32	0.26	0.6557	0.57	0.6011	–0.09	0.7115	0.58	0.3557	0.30	0.7952	0.07	0.7677	0.54	0.4271	0.28	0.8252	0.12	0.6611
rs6813526	88894235	C/T	0.13	1.67	0.04771	2.67	0.06719	–0.24	0.3895	1.91	0.0324	1.89	0.2224	–0.01	0.9617	0.70	0.4694	3.20	0.06073	–0.54	0.1052
rs2728127	88895115	G/A	0.14	1.69	0.03471	2.99	0.03169	–0.29	0.2822	1.93	0.02355	1.93	0.1899	–0.01	0.983	1.28	0.1667	3.68	0.02321	–0.49	0.1255
rs2853744	88896248	T/G	0.07	1.64	0.1167	2.26	0.2209	–0.20	0.5934	3.01	0.00671	4.19	0.03141	–0.35	0.3725	1.34	0.2693	4.95	0.02173	–0.76	0.07875
rs11730582	88896421	T/C	0.46	0.53	0.3454	0.64	0.5377	–0.06	0.8091	0.97	0.1054	0.74	0.5008	0.04	0.8628	0.11	0.8629	–0.66	0.5887	0.24	0.3948
rs11728697	88898941	C/T	0.44	0.24	0.667	0.84	0.4201	–0.18	0.4622	0.92	0.1239	0.94	0.3916	0.00	0.996	0.09	0.8852	–0.14	0.9079	0.12	0.6655
rs6811536	88902405	T/C	0.18	–0.49	0.4734	–1.26	0.303	0.18	0.4957	–1.22	0.09704	–1.38	0.2897	0.08	0.7932	–0.80	0.3165	0.31	0.8282	–0.33	0.2974
rs4754	88902692	C/T	0.43	0.70	0.1951	0.23	0.8117	0.15	0.4827	0.92	0.1085	0.83	0.415	0.01	0.9576	0.84	0.1788	1.12	0.3191	–0.05	0.8477
rs1126616	88903853	T/C	0.43	0.71	0.1878	0.28	0.7698	0.14	0.5191	0.87	0.1253	0.86	0.3963	–0.01	0.9741	0.81	0.1902	1.16	0.2997	–0.07	0.7927
rs1126772	88904186	G/A	0.17	0.69	0.3339	2.29	0.1085	–0.41	0.229	0.90	0.237	0.85	0.5708	0.03	0.9273	0.53	0.5197	0.26	0.8748	0.12	0.7667
rs9138	88904342	C/A	0.43	0.82	0.132	0.26	0.7909	0.18	0.4079	1.00	0.0842	0.84	0.4128	0.03	0.8925	0.85	0.1777	1.08	0.3378	–0.04	0.8841
rs10012150	88905795	C/T	0.17	0.63	0.37	2.16	0.1273	–0.39	0.245	0.88	0.234	0.71	0.6338	0.06	0.8647	0.74	0.3566	0.43	0.7946	0.13	0.7494
rs7685225	88906458	C/T	0.11	0.83	0.3564	3.40	0.03468	–0.66	0.04885	0.12	0.8967	–0.05	0.9769	0.01	0.9745	0.09	0.9341	1.02	0.5889	–0.30	0.4512
rs7675246	88908998	A/G	0.18	–0.72	0.2987	–1.60	0.1913	0.22	0.4219	–1.53	0.03807	–1.97	0.1274	0.15	0.6057	–0.62	0.4351	0.67	0.6378	–0.38	0.2261
Note: Models were adjusted for sex, gestational age, maternal age, marital status, presence of siblings, maternal education, genome-wide principal components 1 and 2, and natural log of second trimester maternal blood Pb level. Alleles are written in the format minor allele/major allele. According to the method proposed by Li and Ji (2005), the *p*-value cut off for this analysis of SNPs in LD to maintain a 5% Type 1 error rate is 0.00568; a single significant result is marked with bold text. Main effects from interaction analysis are provided in Table S2.																					

## Discussion

In this study, we showed that Pb exposure induces an NRF2-mediated transcriptional response in neural stem cells. In particular we identified *SPP1* as a novel NRF2 target gene that is up-regulated by Pb. We further demonstrated the association of a SPP1 genetic polymorphism with cognition development in children. By integrating the global transcriptomic profiling with genetic epidemiology, our study revealed SPP1 up-regulation as a potential mechanistic link between Pb-induced gene expression in NSCs and neurodevelopment in children.

Our study is consistent with other reports of NRF2 activation and up-regulation of NRF2 targets by Pb (Korashy and El-Kadi 2006; Simmons et al. 2011; Yang et al. 2007; Zeller et al. 2010). We note, however, that some transcription profiling studies of Pb-exposed animals (Peterson et al. 2011; Schneider et al. 2012) have not identified NRF2 targets among the top hits. This could be due to the numerous secondary effects of Pb in animals undergoing long-term exposure, as these effects may crowd out the primary cellular transcriptional response. Furthermore, compensatory regulation of the NRF2 pathway may bring down the level of NRF2 activation upon long-term exposure. Although further study is needed to determine the exact mechanisms of Pb’s activation of NRF2, our results implicate NRF2 activation as a new mechanism by which Pb affects NSCs function and neurodevelopment.

We identified *SPP1* as a Pb-induced gene and further demonstrated that *SPP1* is a novel NRF2 target. SPP1 is a pleiotropic extracellular glycoprotein with emerging roles in the brain as a potential neuroprotectant. SPP1 in the brain is up-regulated in several morphological stress conditions including hypoxic ischemia (Albertsson et al. 2014; Chen et al. 2009; Meller et al. 2005), cortical lesion (Chan et al. 2014) and subarchnoid hemorrhage (Topkoru et al. 2013). SPP1 is also induced by a variety of environmental exposures, including cigarette smoke (Shan et al. 2012) and ozone (Bass et al. 2013) in the lung, by chronic manganese exposure in the frontal cortex of manganese exposed nonhuman primates (Guilarte et al. 2008), and by ethanol in human primary neurospheres in culture (Vangipuram et al. 2008). As a secreted protein, SPP1 binds to and activates β3-integrin (β3) to initiate a Focal Adhesion Kinase (FAK) and Protein Kinase B (Akt)-dependent signaling. The β3/FAK/Akt signaling axis is usually anti-apoptotic and pro-proliferative (Fong et al. 2009; Kalluri and Dempsey 2012; Meller et al. 2005; Topkoru et al. 2013). Therefore, SPP1 up-regulation by Pb and secretion from NSCs may induce a compensatory growth and survival response in NSCs and other neural cells.

While this is the first report indicating SPP1 is a direct target of NRF2, a relationship between the two factors has been suggested previously. Consistent with our finding that oxidative stress increases SPP1 expression, exposure of MG63 cells to the oxidative stress inhibitor n-acetylcysteine (NAC) down-regulates SPP1 expression (Kim et al. 2011). In addition, SPP1-mediated signaling through Akt and ERK is suggested to affect migration in glioma cells by activation of NRF2 (Lu et al. 2012), which would suggest a positive feedback loop. The regulatory feedback, however, is complicated by a possible negative feedback loop in which HMOX1, an NRF2 target, suppresses the transcription factor RUNX2, which positively regulates SPP1 expression (Kook et al. 2015). Further studies are needed to untangle the regulatory dynamics and to better understand their implications for Pb-mediated regulation of SPP1 in neurodevelopment.

The role of SPP1 in Pb neurotoxicity is further strengthened by the association of SPP1 variants with cognitive development in the ELEMENT birth cohort. Pb exposure has been repeatedly linked to neurocognitive dysfunction (Fulton et al. 1987; Hu et al. 2006; Needleman et al. 1979, 1996; Wasserman et al. 1997). We identified a SNP, rs12641001, with a statistically significant main effect association with CDI. A second SNP, rs2853744, had a near significant main effect association with LDI. The locations of rs12641001 and rs2853744 upstream of SPP1 suggest a possible role of these SNPs in the regulation of *SPP1* gene expression. Despite our *in vitro* evidence for a neuroprotective role of SPP1 in Pb-treated hNSCs, we did not identify statistically significant interaction between any of the SPP1 SNPs sand Pb exposure. Given that the sample size needed to detect significant gene and environment interactions is in general much larger than that for detecting main effects, it is likely that our ELEMENT cohort was underpowered to identify such interactions. Further epidemiological studies of a larger cohort are needed to test this and to help identify the causal variants in SPP1 that determine the neurodevelopment outcomes in children exposed to Pb.

## Conclusions

The results we reported here reveal that Pb induces an NRF2-mediated transcriptional response, including the up-regulation of a novel NRF2 target SPP1 in NSCs, and that SPP1 genetic polymorphism is associated with neurodevelopment outcomes in children. Our study thus identified SPP1 up-regulation as a potential novel mechanism linking Pb exposure with neural stem cell function and neurodevelopment in children. Further mechanistic studies are needed to elucidate the role of SPP1 and NRF2 activation in modulating the effects of Pb on NSC function and neurodevelopment.

## Supplemental Material

(156 KB) PDF

(2.4 MB) ZIP
