# Supplementary material for: In Vitro Effects of Lead on Gene Expression in Neural Stem Cells and Associations between Up-regulated Genes and Cognitive Scores in Children
Source: Environ Health Perspect. 2016 Aug 26;125(4):721–9. doi: 10.1289/EHP265 (PMC5381979; doi:10.1289/EHP265)

Environ Health Perspect
DOI: 10.1289/EHP265

Note to readers with disabilities: *EHP* strives to ensure that all journal content is accessible to all readers. However, some figures and Supplemental Material published in *EHP* articles may not conform to 508 standards due to the complexity of the information being presented. If you need assistance accessing journal content, please contact ehp508@niehs.nih.gov. Our staff will work with you to assess and meet your accessibility needs within 3 working days.

Supplemental Material

In Vitro Effects of Lead on Gene Expression in Neural Stem Cells and Associations between Upregulated Genes and Cognitive Scores in Children

Peter J. Wagner, Hae-Ryung Park, Zhaoxi Wang, Rory Kirchner, Yongyue Wei, Li Su, Kirstie Stanfield, Tomas R. Guilarte, Robert O. Wright, David C. Christiani, and Quan Lu

Table of Contents

Table S1. Primers Used in Study

Table S2. Characteristics of the ELEMENT cohort

Figure S1. Histone H3 ChIP followed by PCR amplification of the putative SPP1 ARE. NRF2 was activated by KEAP1 knockdown (H3 si). NT-siRNA was transfected into control cells (H3 NT). Following H3 ChIP, qPCR was done to measure the presence of the SPP1 ARE.

Additional File

Supplemental Code and Data ZIP File

Excel File Table S1. Full results from the differential expression analysis

Table S1. Primers Used in Study

Target	Application	Forward	Reverse
DIO3OS	QPCR	GCTACAGGCTTCAGCAGGTC	CCCCAGACAGCAGAGAAATC
F2RL2	QPCR	GCAAAGCCAACCTTACCCATT	GAGGTAGATGGCAGGTATCAGT
FTL	QPCR	CAGCCTGGTCAATTTGTACCT	GCCAATTCGCGGAAGAAGTG
HMOX1	QPCR	GGCAGAGGGTGATAGAAGAGG	AGCTCCTGCAACTCCTCAAA
LUCAT1	QPCR	CGATCTCGGTTCACTGCAA	AGCAAACAGCAAGTTGGATTC
NQO1	QPCR	GAAGAGCACTGATCGTACTGGC	GGATACTGAAAGTTCGCAGGG
OSGIN1	QPCR	CGAGGGTGTCGAGAAGGTG	TCCACGTCAATGGGGTTCCT
SPP1	QPCR	GGAGTTGAATGGTGCATACAAGG	CCACGGCTGTCCCAATCAG
KEAP1	QPCR	CTGGAGGATCATACCAAGCAGG	GGATACCCTCAATGGACACCAC
NRF2	QPCR	TCCAGTCAGAAACCAGTGGAT	GAATGTCTGCGCCAAAAGCTG
SPP1-a	QPCR	ATCTCCTAGCCCCACAGAAT	CATCAGACTGGTGAGAATCAT
SPP1-b	QPCR	ATCTCCTAGCCCCAGAGAC	AAAATCAGTGACCAGTTCATCAG
SPP1-c	QPCR	TGAGGAAAAGCAGAATGCTG	GTCAATGGAGTCCTGGCTGT
NQO1 ARE	ChIP-QPCR	CCCTTTTAGCCTTGGCACGAAA	TGCACCCAGGGAAGTGTGTTGTAT
SPP1 ARE	ChIP-QPCR	ATAGGTAGGCTGGGCGATTT	GTGGTTCTGAATTCCGCTGT
GAPDH	QPCR	GGAAACTGTGGCGTGATG	AGTGAGCTTCCCGTTCAG

Table S2. Characteristics of the ELEMENT cohort

Trait	Cohort
Number of participants	462
Sex (male/female)	245/217
Mean gestational age in weeks (sd)	38.38 (1.65)
Mean maternal age in years at birth (sd)	27.81 (5.51)
Percentage of mothers married	80.3%
Percentage of children with siblings	53.0%
Percentage of mothers who completed high school education	58.4%
Mean second trimester maternal blood Pb level in ug/dL (sd)	3.76 (2.62)
Mean Raw Cognitive Development Index at ~24 months (sd)	92.48 (8.33)
Mean Raw Language Development Index at ~24 months (sd)	89.76 (9.08)
Mean Raw Psychomotor Development Index at ~24 months (sd)	93.95 (10.58)

Figure S1. Histone H3 ChIP followed by PCR amplification of the putative SPP1 ARE. NRF2 was activated by KEAP1 knockdown (H3 si). NT-siRNA was transfected into control cells (H3 NT). Following H3 ChIP, qPCR was done to measure the presence of the SPP1 ARE.

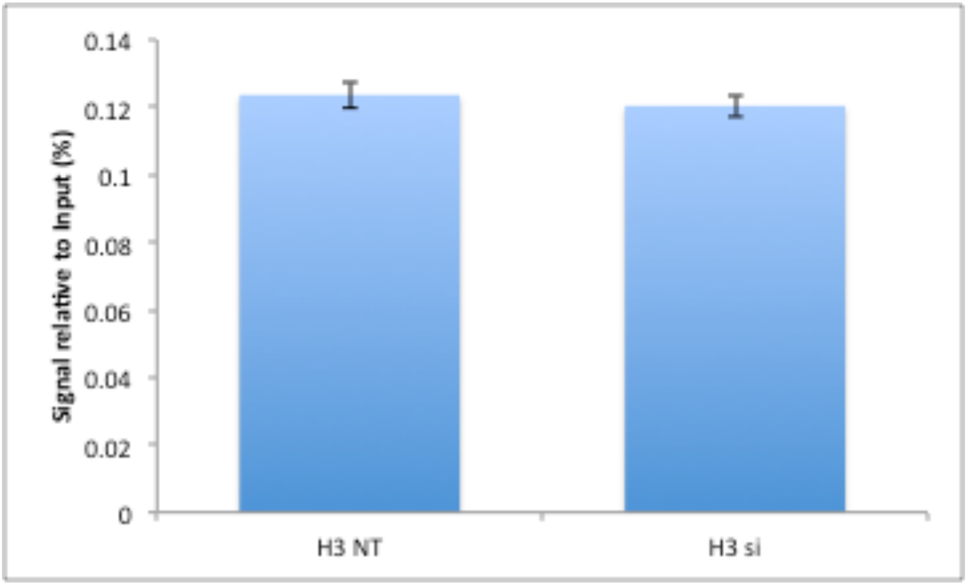

Supplement: (156 KB) PDF [file EHP265.s001.acco.pdf]
